# Potential of Kidney Exchange Programs (KEPs) in Japan for Donor-Specific Antibody-Positive Kidney Transplants: A Questionnaire Survey on KEPs and a Multi-Institutional Study Conducting Virtual Cross-Matching Simulations

**DOI:** 10.3390/jcm14176122

**Published:** 2025-08-29

**Authors:** Taihei Ito, Miki Ito, Naohiro Aida, Kei Kurihara, Akihiro Terao, Yoshihiko Watarai, Mitsuru Saito, Keizo Kaku, Daisuke Ishii, Satoshi Sekiguchi, Tatsuo Yoneda, Kohei Unagami, Masayuki Tasaki, Hitoshi Iwamoto, Motoo Araki, Kazuhiro Takahashi, Kazuaki Yamanaka, Mikio Sugimoto, Kouhei Nishikawa, Chikashi Seto, Masaki Muramatsu, Toshihiro Asai, Daiki Iwami, Yasutoshi Yamada, Shigeyoshi Yamanaga, Tomonori Komatsu, Masayoshi Miura, Takahiro Nohara, Michihiro Maruyama, Yuki Miyauchi, Toshiaki Tanaka, Michio Nakamura, Kiyohiko Hotta, Takashi Kenmochi

**Affiliations:** 1 Department of Transplantation and Regenerative Medicine, School of Medicine, Fujita Health University, Dengakugakubo 1-98, Kutsukakecho, Toyoake 470-1192, Japan; peach.apricot.0925@gmail.com (M.I.); n-aida@fujita-hu.ac.jp (N.A.); k.k.530504@gmail.com (K.K.); akihiro.terao@fujita-hu.ac.jp (A.T.); kenmochi@fujita-hu.ac.jp (T.K.); 2 Department of Transplant Surgery, Japanese Red Cross Aichi Medical Center Nagoya Daini Hospital, Aichi 466-8650, Japan; watarai@nagoya2.jrc.or.jp; 3 Division of Blood Purification, Akita University Hospital, Akita 010-8543, Japan; urosaito@gmail.com; 4 Department of Surgery and Oncology, Graduate School of Medical Sciences, Kyushu University, Fukuoka 812-8582, Japan; k-kaku@kyudai.jp; 5 Department of Urology, Kitasato University of Medicine, Kanagawa 252-0375, Japan; ishiiccf@gmail.com; 6 Transplantation Surgery, Japan Community Healthcare Organization Sendai Hospital, Miyagi 981-3281, Japan; sekiguchi12@me.com; 7 Unit of Dialysis, Department of Urology, Nara Medical University, Nara 634-8522, Japan; uro-yone@naramed-u.ac.jp; 8 Organ Transplant Medicine, Tokyo Women’s Medical University, Tokyo 162-8666, Japan; unagami.kohei@twmu.ac.jp; 9 Division of Urology, Department of Regenerative & Transplant Medicine, Graduate School of Medical and Dental Sciences, Niigata University, Niigata 951-8510, Japan; masa1214@med.niigata-u.ac.jp; 10 Department of Kidney Transplantation Surgery, Tokyo Medical University Hachioji Medical Center, Tokyo 193-0998, Japan; hitoiwa@tokyo-med.ac.jp; 11 Department of Urology, Okayama University Graduate School of Medicine, Dentistry and Pharmaceutical Sciences, Okayama 700-8558, Japan; motoosh@md.okayama-u.ac.jp; 12 Department of Gastrointestinal and Hepatobiliary Pancreatic Surgery, University of Tsukuba, Ibaraki 305-8575, Japan; kazu1123@md.tsukuba.ac.jp; 13 Department of Urology, Osaka University Graduate School of Medicine, Osaka 565-0871, Japan; yamanaka@uro.med.osaka-u.ac.jp; 14 Department of Urology, Faculty of Medicine, Adrenal Surgery and Renal Transplantation, Kagawa University, Kagawa 761-0793, Japan; sugimoto.mikio@kagawa-u.ac.jp; 15 Department of Nephro-Urologic Surgery and Andrology, Mie University Graduate School of Medicine, Mie 514-8507, Japan; kouheini@clin.medic.mie-u.ac.jp; 16 Department of Urology, Toyama Prefectural Central Hospital, Toyama 930-0194, Japan; chiseto11@fd6.so-net.ne.jp; 17 Department of Nephrology, Toho University Faculty of Medicine, Tokyo 143-8541, Japan; masaki.muramatsu@med.toho-u.ac.jp; 18 Department of Kidney Transplant and Dialysis, Osaka City General Hospital, Osaka 534-0021, Japan; demetriosanna@yahoo.co.jp; 19 Division of Renal Surgery and Transplantation, Department of Urology, Jichi Medical University, Tochigi 329-0498, Japan; iwamidaiki@ybb.ne.jp; 20 Department of Blood Purification, Kagoshima University Hospital, Kagoshima 890-8520, Japan; giosyamada@gmail.com; 21 Department of Transplant Surgery, Japanese Red Cross Kumamoto Hospital, Kumamoto 861-8502, Japan; yama335@gmail.com; 22 Department of Urology, Chukyo Hospital, Japan Community Healthcare Organization, Aichi 457-8510, Japan; komatsutomonori@gmail.com; 23 Department of Renal Transplantation Surgery and Urology, Sapporo Hokuyu Hospital, Hokkaido 003-0006, Japan; uromasa@mac.com; 24 Department of Urology, Kanazawa University Hospital, Kanazawa 920-8641, Japan; t_nohara704@yahoo.co.jp; 25 Department of Frontier Surgery, Chiba University School of Medicine, Chiba 260-8670, Japan; michi-maruyama@chiba-u.jp; 26 Department of Urology, Ehime University, Ehime 791-0295, Japan; miyauchi.yuki.mf@ehime-u.ac.jp; 27 Department of Urology, Sapporo Medical University, Hokkaido 060-8543, Japan; zappa@pop12.odn.ne.jp; 28 Department of Transplant Surgery, Tokai University School of Medicine, Kanagawa 259-1193, Japan; m_nakamura@tokai.ac.jp; 29 Department of Renal and Genitourinary Surgery, Faculty of Medicine, Hokkaido University, Hokkaido 060-8648, Japan; hotta1125@mac.com

**Keywords:** kidney transplantation, donor-specific antibodies, kidney exchange program, virtual cross-matching

## Abstract

**Objectives:** To clarify the need for a kidney exchange program (KEP) in Japan by conducting a questionnaire survey on KEPs and simulated KEPs by virtual cross-matching based on past cases of transplantation avoidance. **Methods:** In addition to the content regarding KEPs, an electronic survey was conducted to investigate the number of cases of kidney transplant abandonment due to “immunological” reasons over the past 10 years (2012–2021). Virtual cross-matching was conducted to simulate the feasibility of avoiding immunological risks and enabling kidney transplantation in patients who were previously unable to undergo the procedure. **Results**: The survey received responses from 107 facilities (response rate: 81.7%). In response to the question about the necessity of a KEP in Japan, 71 facilities (66.4%) indicated that KEPs are necessary. In addition, 251 living-donor kidney transplants were abandoned for “immunological” reasons over the past decade (2012–2021). Among the 80 pairs for which detailed information was available, virtual cross-matching simulations showed that 37/80 pairs (46.3%) were donor-specific antibody (DSA)-negative for blood type-matched combinations, and 41/80 pairs (51.3%) were DSA-negative for blood type-incompatible transplants. **Conclusions:** The need for a KEP in Japan and its potential usefulness were demonstrated.

## 1. Introduction

In Japan, more than 90% of kidney transplants are performed using living donors, and approximately 30% of these are blood type-incompatible transplants [1]. Furthermore, patients who are positive for donor-specific antibodies (DSAs) undergo desensitization therapy prior to kidney transplantation, and the outcomes are considered relatively favorable [2,3,4,5,6]. Depending on the cross-matching results, there are highly sensitized recipient candidates for whom transplantation must be avoided; however, the frequency of such cases remains unclear. In addition, kidney exchange programs (KEPs) for blood type-incompatible or DSA-positive patients have gained popularity worldwide, and their safety and efficacy have been reported [7,8,9,10,11,12,13,14].

In Japan, one paired kidney exchange transplant was performed in 2003 to avoid a blood type-incompatible transplant [15]. At that time, the following statement was issued by the Japan Society for Transplantation on 11 June 2004 [16].

(1) While recent advances in transplantation technology have improved the outcomes of kidney transplantation under these conditions, many transplant specialists believe that there is never such a great medical necessity that would require donor exchange to perform transplantation.
(2) This is based on the intention to donate one’s own organs for the benefit of one’s relatives and does not violate the ethical guidelines of the Japan Society for Transplantation.
(3) Donor exchange kidney transplantation involves major medical and ethical issues and should be conducted under the ethical review of each facility on a case-by-case basis. Therefore, donor exchange kidney transplantation should not be promoted through social systems such as donor exchange networks.

Twenty years have passed since the above statement, and immunological risk assessment methods have since improved. Pre-transplantation desensitization therapy options, such as rituximab and intravenous immunoglobulin, are now available in Japan and are covered by insurance. However, is there really an absolute medical need for a KEP? If Japan has a need, what are the potential benefits of implementing a KEP?

The purpose of this study was to assess the need for a KEP by conducting a questionnaire survey of kidney transplantation facilities in Japan. Based on the survey results, the second purpose of the study was to conduct a feasibility study through virtual cross-matching simulation, collect information from highly sensitized recipient candidates who had previously abandoned living kidney transplantation due to their immunological risks, and to determine whether implementing a KEP can mitigate immunological risks and increase transplantation chances.

## 2. Materials and Methods

### 2.1. Research to Identify the Need for a KEP (Primary Research)

An electronic survey using Google Forms was conducted from December 2021 to February 2022 to identify the need for a KEP in Japan. A total of 131 kidney transplantation facilities across the nation were asked to provide responses to the questionnaire. The survey included the following details:

1. The “immunological” criteria precluding kidney transplantation.
2. Number of living-donor kidney transplants abandoned for “immunological” reasons over the last decade (2012–2021) and the underlying causes.
3. What policy would be preferred in cases of abandonment mentioned in point 2 above?
4. Do you think that a KEP for highly sensitive cases is necessary in Japan?
5. “Immunological” scope of transplant eligibility if a KEP is established in Japan.
6. Problems with KEPs.
7. “Ethical” preoperative preparation for KEPs.
8. “Transplant surgery method” in KEPs.

### 2.2. Simulation of Virtual Cross-Matching Through Multi-Institutional Collaboration (Secondary Research)

A virtual cross-matching simulation was conducted to assess whether KEPs could mitigate immunological risks and make kidney transplantation feasible. A multi-institutional collaborative research project titled “survey to elucidate the need for a KEP” was conducted with the cooperation of 35/251 transplant facilities that reviewed 251 cases of living-donor kidney transplants abandoned for immunological reasons over the past 10 years (2012–2021).

Donor (age, sex, blood type, relationship to the recipient, past medical history, serum creatinine level (s-Cre), and estimated glomerular filtration rate (eGFR)) and recipient patient information (age, sex, blood type, and immunological test results: HLA typing, direct cross-match, flow cytometry cross-match, flow PRA, and anti-HLA antibody single antigen identification tests) from 117 pairs were collected using the REDCap system [17,18]. Information on the 137 transplantation abandonment cases was not collected because these cases were from institutions not participating in this multi-institutional collaborative research project. The background data of these donor and recipient candidates were compared with those of living-donor kidney transplant donors and recipients in Japan between 2012 and 2021. These data were collected from annual reports [19,20,21,22,23,24,25,26,27,28] and aggregated by the authors.

**Figure 1:**
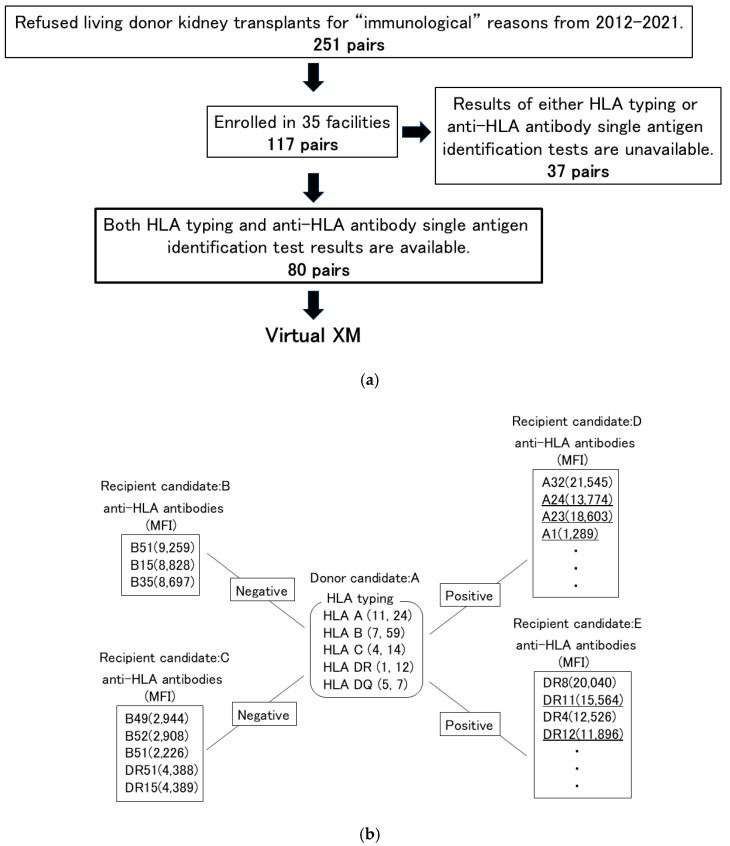
Secondary research: Multi-institutional KEP simulation with virtual cross-matching. Of the 251 pairs who refused living-donor kidney transplants owing to immunological reasons, 117 pairs from 35 facilities were enrolled in the multi-institutional study. However, detailed information on anti-HLA antibody using single antigen identification tests was obtained for 80 pairs, and simulation was possible through virtual cross-matching (**a**). Example of virtual cross-match (**b**). Bold underlines indicate donor-specific antibodies, and thin underlines indicate potentially cross-reactive anti-HLA antibodies.

Virtual cross-matching was feasible for 80/117 pairs because cases screened for all anti-HLA DSAs and non-DSAs by the Luminex assay were included (Figure 1a).

### 2.3. Example of Virtual Cross-Matching

Participants with an MFI value ≥1000 were considered positive for anti-HLA antibodies. Virtual cross-matching was performed using HLA typing by serotype, and those with potential cross-reactivity based on the cross-reactivity map [29] were considered DSA-positive (Figure 1b). Accordingly, we compiled the number of candidate recipients who would be DSA-negative for each individual donor. In the present study, overlapping cases were not considered as donor candidates to determine how many recipient candidates were DSA-negative and transplant-eligible. A physician and a coordinator performed virtual cross-matching and double-checked the results.

### 2.4. Statistical Analyses

All statistical analyses were performed using EZR software version 1.68 (freely available from the Saitama Medical Center, Jichi Medical University), which extends the functionality of R and R Commander version 4.20 [30]. Student’s *t*-test and Fisher’s exact test were used, with statistical significance defined as a *p*-value of ≤ 0.05.

### 2.5. Ethical Considerations

The present study was conducted with full consideration of ethical aspects, adhering to the Declaration of Helsinki’s “Ethical Principles for Medical Research Involving Human Subjects” (revised in October 2013) and the ethical guidelines of the Japan Society for Transplantation (revised in September 2021). Furthermore, approval was obtained from the Ethical Review Board of Fujita Health University for the primary and secondary research, respectively (M21-462, HM22-392). The secondary research was a multi-institutional collaborative study. Therefore, the Ethical Review Board of Fujita Health University conducted a central review and provided approval. Afterward, permission to conduct the study was obtained from the director of each institution.

## 3. Results

### 3.1. Research to Identify the Need for a KEP (Primary Research)

**Figure 2:**
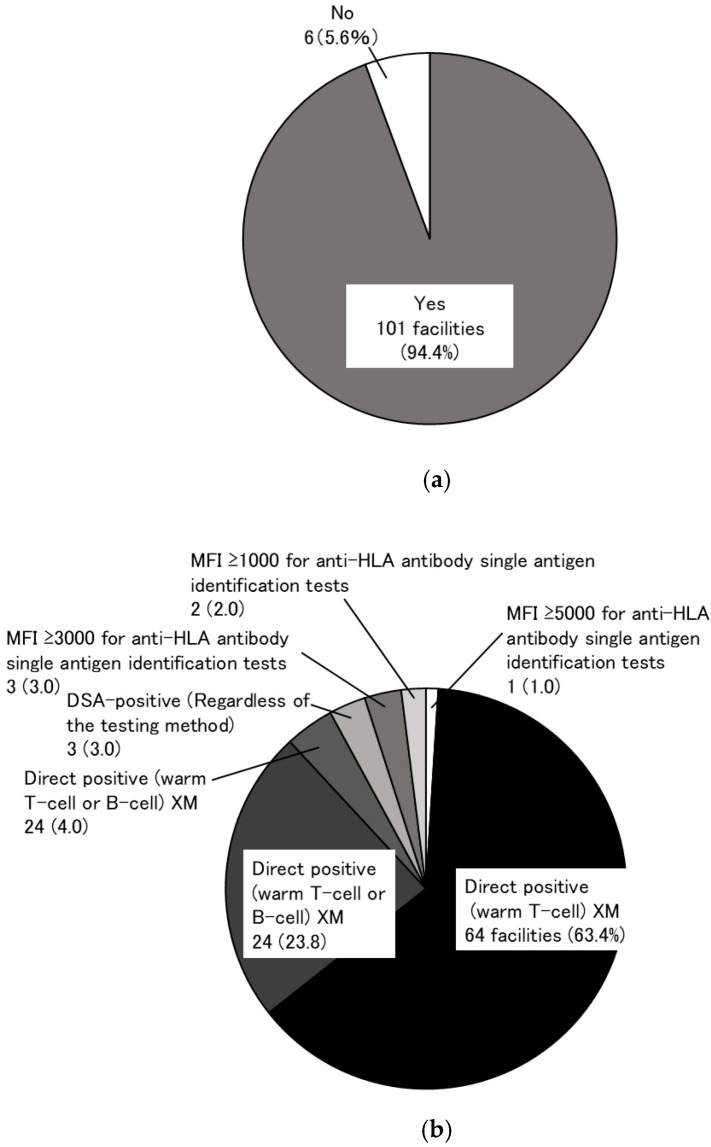
“Immunological” criteria precluding kidney transplantation. The presence or absence of institutional criteria for “immunological” test results that preclude kidney transplantation (**a**) and specific criteria (**b**). In 64 facilities (63.4%), direct positive warm T-cell XM was the immunological criterion that precluded transplantation.

**Figure 3:**
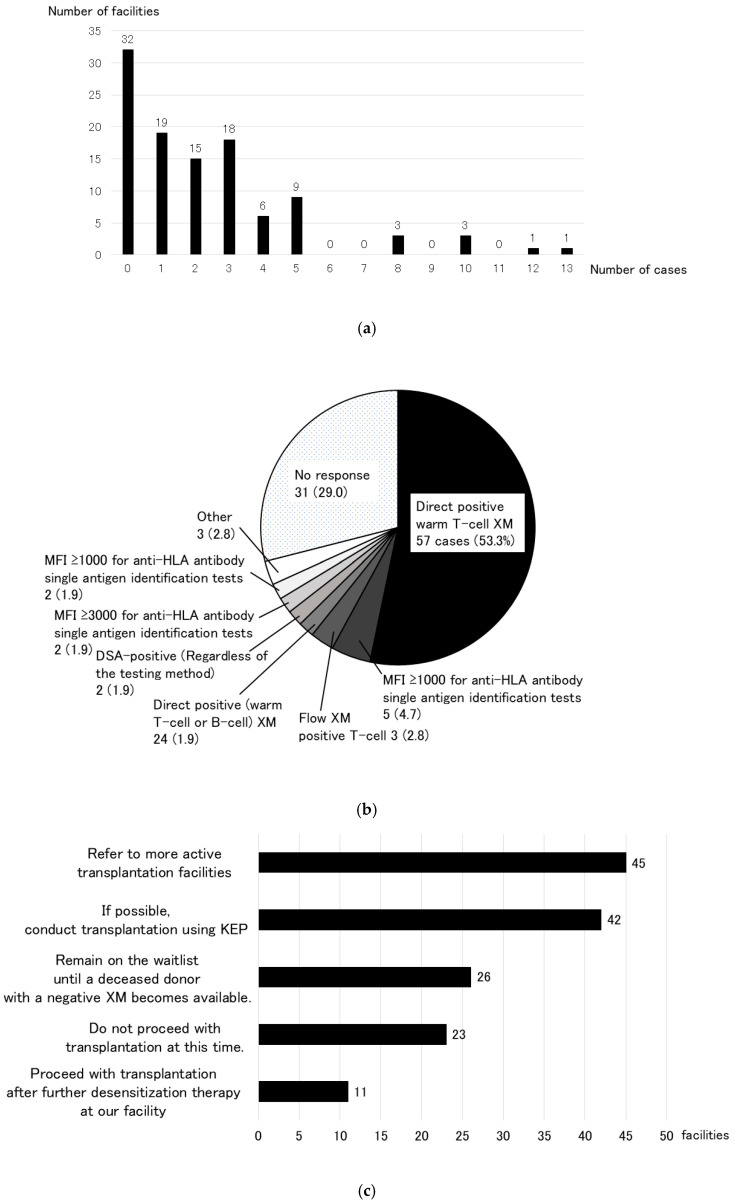
Number of cases over the past 10 years (2012–2021) in which living-donor kidney transplantation was abandoned due to “immunological” reasons (**a**), a breakdown of the reasons for this decision (**b**), and methods considered appropriate for these cases (**c**). In the past decade (2012–2021), 251 living-donor kidney transplant candidates had to abandon transplantation due to “immunological” reasons. Seventy-five of the one hundred-and-seven facilities (70.1%) had abandoned at least one kidney transplant.

The survey received responses from 107 facilities (response rate: 81.7%). To the question, “Are there any criteria for “immunological” test results that would preclude kidney transplantation at your facility?”, 101 facilities (94.4%) responded “yes” (Figure 2a). Of these, 64 facilities (63.4%) cited direct positive warm T-cell cross-match (XM) as an immunological contraindication for transplantation (Figure 2b). A total of 251 living-donor kidney transplant candidates were declined for “immunological” reasons during the past 10 years (2012–2021) at 107 facilities that responded to the questionnaire (Figure 3a). Seventy-five of the one hundred-and-seven facilities (70.1%) had abandoned at least one kidney transplant for immunological reasons in the past decade. The most common immunological reason for this finding was direct positive warm T-cell XM in 57 cases (53.3%) (Figure 3b). In response to the question, “What do you think would be the best approach for these transplant abandonment cases?” (multiple answers allowed), the most frequent answer at 45 facilities (42.1%) was to refer the patients to a more active transplant facility. However, the second-most frequent response at 42 facilities (39.3%) was to use a KEP for transplantation if possible (Figure 3c).

**Figure 4:**
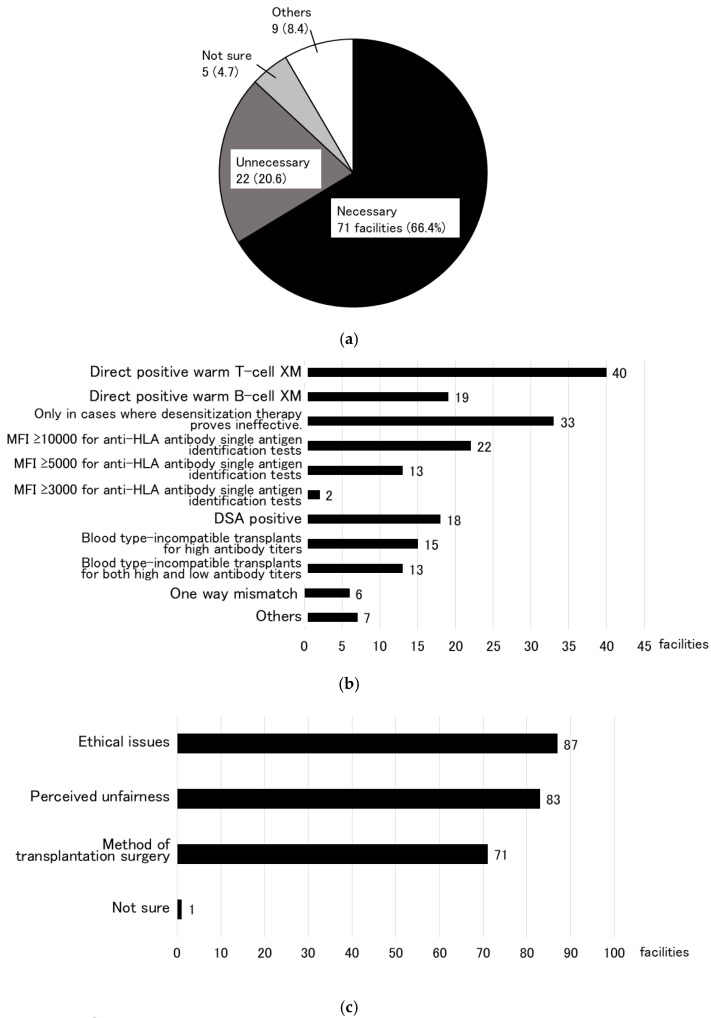

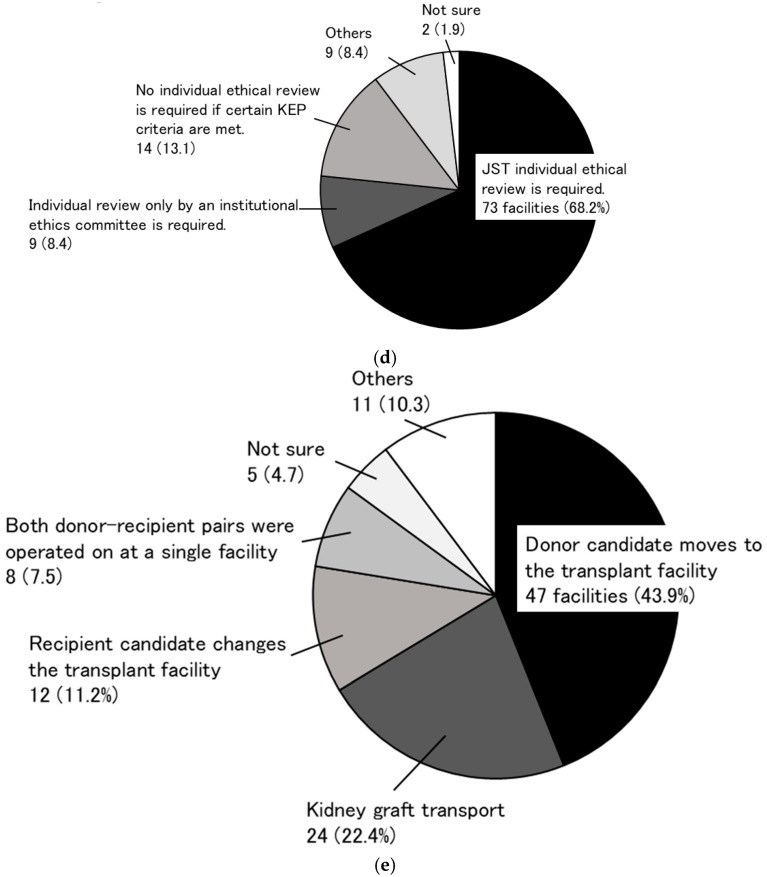
Need for a KEP in Japan (**a**), “immunological” criteria for and transplant eligibility if a KEP is established (**b**), issues (**c**), “ethical” considerations in preoperative preparation (**d**), and transplant surgical techniques (**e**). In the future, 71 facilities (66.4%) indicated that a KEP is needed in Japan.

When asked whether KEPs are necessary in Japan in the future, 71 facilities (66.4%) responded that it is, while 22 facilities (20.6%) responded that it is unnecessary (Figure 4a). Regarding the immunological criteria for establishing and implementing KEP in Japan, the most common response at 40 facilities (37.4%) was direct positive warm T-cell XM, followed by 33 facilities (30.8%) that stated that KEP should only be implemented when desensitization therapy is unsuccessful (Figure 4b). Other responses included DSA positivity at 18 facilities (16.8%), blood type-incompatible transplants (15 facilities (14.0%) for high antibody titers and 13 facilities (12.1%) for both high- and low-antibody titers), and one-way mismatches (6 facilities (5.6%)).

When asked about problems with KEPs (multiple responses allowed), 87 facilities (81.3%) cited ethical issues, 83 facilities (77.6%) perceived unfairness, and 71 facilities (66.4%) cited the method of transplantation surgery, which were considered issues that need to be resolved in the future when introducing a KEP in Japan (Figure 4c). Regarding ethical issues, 73 facilities (68.2%) responded that an individual ethical review is required by the Japanese Society of Transplantation, and 9 facilities (8.4%) responded that an individual review by an institutional ethics committee is required. This is far more than the 14 facilities (13.1%) that responded that individual ethical review is not required if certain KEP criteria are met. Many facilities thought that some type of ethical review was necessary (Figure 4d). Regarding the surgical method of KEP, 47 facilities (43.9%) responded that the donor candidate moves to the transplant facility to undergo surgery, and 24 facilities (22.4%) responded that the recipient candidate changes the transplant facility to undergo the transplantation at the hospital where the donor candidate is located (Figure 4e). Only 12 facilities (11.2%) permitted kidney graft transport, similar to the method used in the United States.

### 3.2. Simulation Using Virtual Cross-Matching (Secondary Research)

#### 3.2.1. Donor Candidate Background

A total of 117 donor–recipient candidate pairs were included in the secondary study, comprising 80 in the virtual cross-match group and 37 in the exclusion group. Table 1 shows a comparison of donor backgrounds. The mean donor age was 56.5 ± 12.1 years overall, with no significant difference between the virtual cross-match group (56.3 ± 11.0 years) and the exclusion group (56.9 ± 14.5 years; *p* = 0.815). The age distribution differed significantly between groups (*p* = 0.026), with only one donor aged 20–29 years in the exclusion group and none in the virtual cross-match group. The overall sex ratio was male-dominant (81 males and 35 females), with no significant difference between groups (virtual: 58 males/22 females; excluded: 23 males/13 females; *p* = 0.386). Spouses were the most common donor–recipient relationship (70 husbands, 11 wives), followed by parents, siblings, and others. No significant difference was found in relationship distribution (*p* = 0.131). The prevalence of hypertension was significantly higher in the virtual cross-match group than in the exclusion group (17 vs. 3; *p* = 0.018). Diabetes was more prevalent in the virtual group (9 cases vs. 1 case), though not significantly (*p* = 0.058). s-Cre was comparable between groups (0.82 ± 0.14 vs. 0.93 ± 1.15 mg/dL; *p* = 0.430), while estimated GFR tended to be lower in the virtual group (72.6 ± 12.3 vs. 78.3 ± 18.4 mL/min/1.73 m^2^), though this was not statistically significant (*p* = 0.074).

Among the 15,583 living-donor kidney transplants performed in Japan from 2012 to 2021, 14,354 were registered in the Japan Kidney Transplant Registry. Compared to this broader cohort, significant differences were observed in sex distribution (*p* < 0.001) and donor–recipient relationship types (*p* < 0.001). Hypertension and diabetes prevalence also differed significantly (*p* = 0.890 and *p* = 0.014, respectively), reflecting distinct clinical characteristics in the virtual cross-match group.

#### 3.2.2. Recipient Candidate Background

Recipient background characteristics were similarly compared between groups (Table 2). The overall mean recipient age was 52.1 ± 10.6 years, with no significant difference between the virtual cross-match (51.2 ± 10.7) and exclusion groups (53.9 ± 10.3; *p* = 0.201). Age distribution was not significantly different (*p* = 0.166). There was a female predominance in both groups (overall: 22 men, 95 women), with no significant difference (virtual: 15 men/65 women; excluded: 7 men/30 women; *p* = 1.000). Blood type compatibility was similar (65 matches vs. 49 mismatches; *p* = 0.839).

The HLA mismatch distribution was significantly different between groups (*p* = 0.024), with most candidates having 3–5 mismatches. No significant difference was observed in T-cell direct cross-match results (overall positive–negative ratio, 50:54; *p* = 0.669). However, a statistically significant difference was noted in B-cell direct cross-match results (overall positive–negative ratio, 58:39; *p* = 0.044). Flow cytometry cross-match testing for both T- and B-cells showed no significant differences between the virtual cross-match and exclusion groups. Panel-reactive antibody (PRA) Class I and Class II levels, assessed by flow cytometry, also did not differ significantly between the groups (Class I: *p* = 0.847; Class II: *p* = 0.101). As all cases were immunologically high-risk, the donor-specific antibody (DSA) positivity rate was 100% in both groups, by definition.

When compared with recipients of living-donor kidney transplants performed in Japan between 2012 and 2021, significant differences were observed in gender distribution (male/female = 9192:5162; *p* < 0.001), blood type compatibility (compatible/incompatible = 8671:4033; *p* = 0.029), HLA mismatch distribution (*p* = 0.024), and cross-match positivity rates (direct T- and B-cell warm cross-match, and flow cytometry T- and B-cell cross-match; *p* < 0.001). These findings indicate that immunologically high-risk recipients differ significantly from the general living-donor kidney transplant population in several key immunological and demographic parameters.

#### 3.2.3. Simulation 1

**Figure 5:**
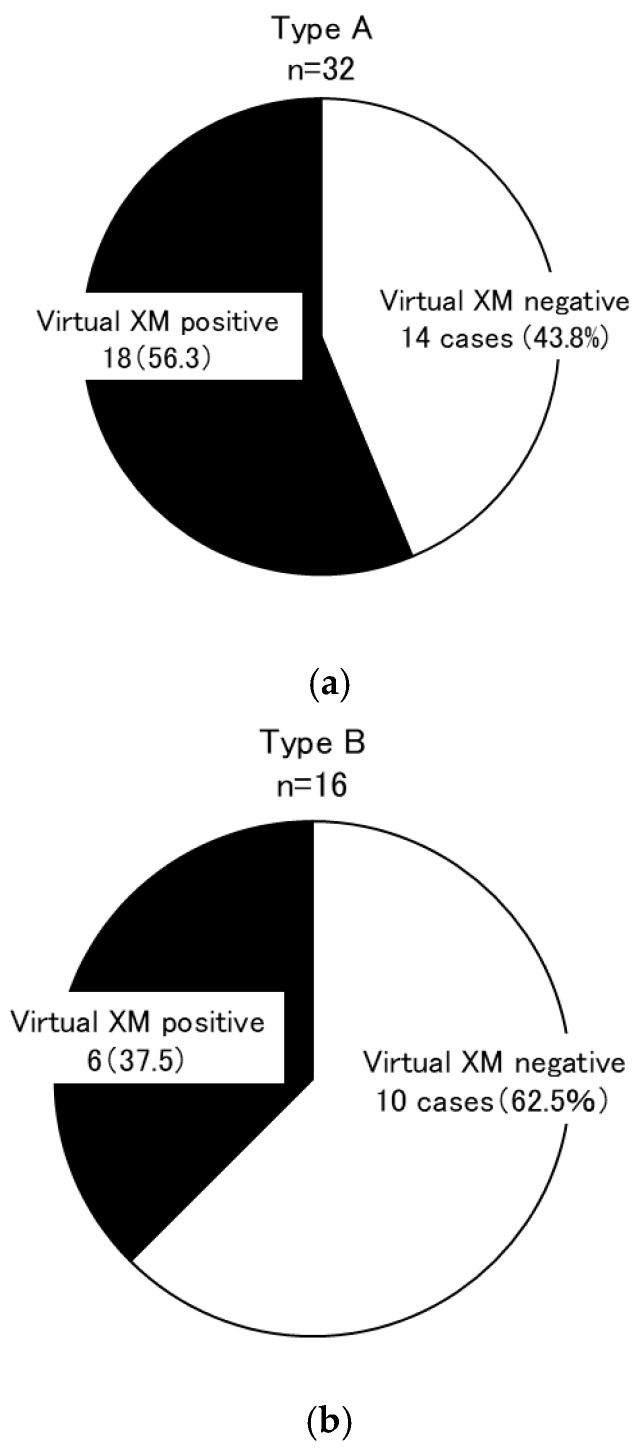

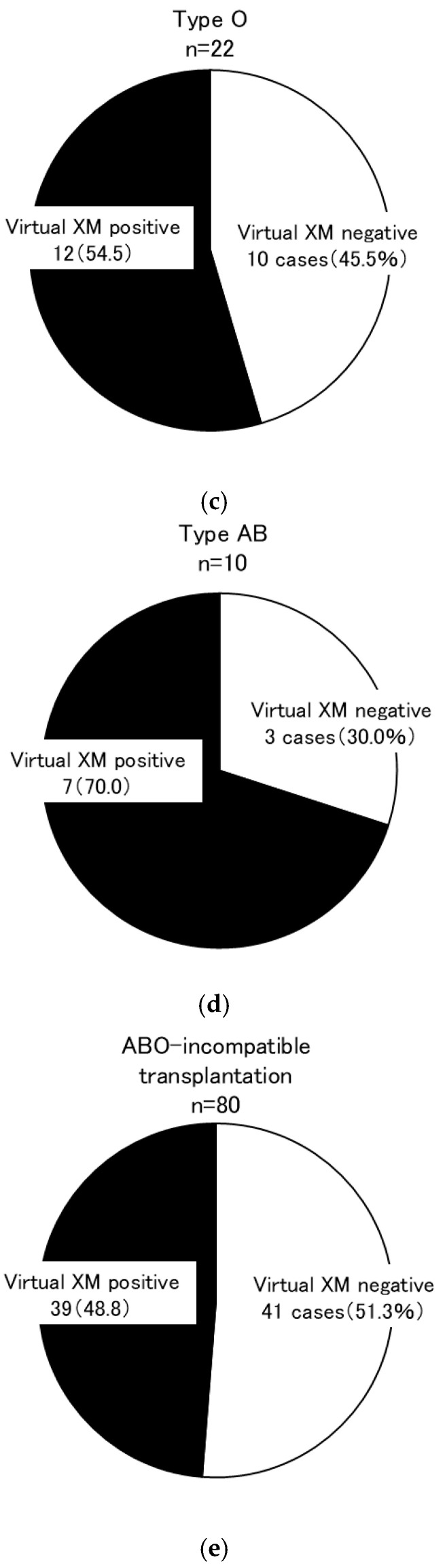
KEP Simulation via virtual cross-matching. We used virtual cross-matching to examine combinations that were negative for DSA, with a mean fluorescence intensity cutoff value of 1000 or less for the anti-HLA antibody single antigen identification test to define negativity. DSA-negative combinations were established in 14/32 pairs for type A (43.8%) (**a**), 10/16 pairs for type B (62.5%) (**b**), 10/22 pairs for type O (45.5%) (**c**), and 3/10 pairs for type AB (30.0%) (**d**). Overall, a blood type-matched kidney transplant combination that avoided DSA was possible in 37/80 pairs (46.3%). In 41/80 pairs (51.3%) of blood type-incompatible transplants, transplantation was possible while avoiding DSA (**e**).

A mean fluorescence intensity cutoff value of less than 1000 for the anti-HLA antibody single antigen identification test was considered negative, and combinations of blood type-matched donor and recipient candidates that were DSA-negative were analyzed using virtual cross-matching. DSA-negative combinations were identified in 14/32 pairs of type A (43.8%) (Figure 5a), 10/16 pairs of type B (62.5%) (Figure 5b), 10/22 pairs of type O (45.5%) (Figure 5c), and 3/10 pairs of type AB (30.0%) (Figure 5d). Overall, a blood type-matched kidney transplant combination that successfully avoided DSA could be created in 37/80 pairs (46.3%).

#### 3.2.4. Simulation 2

In Japan, approximately 30% of all living-donor kidney transplants are blood type-incompatible, and the outcomes of these transplants are comparable to those of compatible transplants [1,31]. Although preoperative desensitization therapy is necessary to create further transplant opportunities, kidney transplant combinations that avoid DSA in blood type-incompatible transplants were explored, and transplantation was feasible in 41/80 pairs (51.3%) (Figure 5e). Simulation of one-on-one paired KEP with blood type-incompatible transplants resulted in 21 pairs of transplants.

## 4. Discussion

In Japan, there are no legal regulations regarding the selection of donors for living-donor kidney transplants. However, ethical guidelines of the Japan Society for Transplantation state that only relatives within the sixth degree of consanguinity or in-laws within the third degree of relationship can ethically donate a kidney. Donation from donor candidates who do not meet these criteria (e.g., friends, common-law couples, and same-sex partners) requires ethical reviews by the institution and the Japan Society for Transplantation on a case-to-case basis. Previous paired kidney exchange transplants performed individually did not undergo ethical reviews by the Japan Society for Transplantation. However, they eventually concluded that there were no ethical concerns with these cases. At the same time, they found no urgent necessity for the establishment of nationwide KEPs. This study aimed to challenge the two-decade-old view of the Japan Society for Transplantation: are KEPs still unnecessary in Japan?

The primary findings of this study revealed that 66.4% of kidney transplantation facilities in Japan believe that a KEP is necessary. Furthermore, 251 pairs of living-donor kidney transplants were abandoned for “immunological” reasons in Japan over the 10-year period from 2012 to 2021. In addition, more than 70% of the facilities responding to the survey had experienced at least one pair of transplant abandonment. Moreover, the secondary research results from virtual cross-matching revealed that DSA-negative combinations can be created and transferred in approximately half of the cases studied. We believe these results strongly demonstrate the need for a KEP in Japan and the potential benefits of its establishment.

The number of kidney transplants in Japan is 1700–2000 per year, approximately 90% of which are living-donor kidney transplants. A total of 15,283 living-donor kidney transplants were performed from 2012 to 2021, for which the present simulation was conducted [19,20,21,22,23,24,25,26,27,28]. Of these, 251 patients abandoned transplantation due to high immunological risks, corresponding to 1.6% of all the patients who underwent transplantation. The secondary research showed the possibility that KEP construction could lead to identification of DSA-negative donor candidates in approximately 50% of these highly sensitized recipient candidates. The one-on-one paired KEP simulation including blood type-incompatible transplants resulted in 21 pairs (26.3%). Expansion of the KEP donor pool to include additional donor candidates not considered in this study is expected to create further transplantation opportunities where immunological risks are avoided. In the secondary study population, the majority of individuals who underwent virtual cross-match testing were women. This suggests a potential for sensitization due to previous pregnancies, sexual intercourse, or childbirth, as most of the donor candidates were their husbands.

When comparing the background characteristics of the virtual cross-match group with those of kidney transplant recipients from the same period, the recipient candidates in the virtual cross-match group, as expected, exhibited significantly higher immunological risk. Conversely, statistical testing was not performed to compare donor age and renal function due to the unavailability of detailed variation data for donors from the national transplant cohort. However, the age distribution histograms between the two groups showed no notable differences. Although the s-Cre level was slightly higher in the virtual cross-match group, this donor cohort was still considered suitable for living kidney donation in terms of age and renal function—provided that immunological risks could be appropriately managed or avoided.

Nationwide KEPs for DSA-positive, immunologically high-risk living-donor kidney transplant recipients are being conducted in various Western countries, including the United States [10,32,33,34,35,36], the United Kingdom [37], Germany [38,39], Spain [40], the Netherlands [41], Australia, and New Zealand [42]. This includes efforts to avoid blood type-incompatible transplants [41], as well as the implementation of blood type-incompatible transplants to prevent high-sensitization DSA, as explored in this study [42,43]. Brazil and other countries are considering the introduction of KEP in the future to expand transplantation opportunities for patients with kidney failure [44]. Flechner et al. [10] reported that donor exchange kidney transplantation in the United States, specifically through the National Kidney Registry, is steadily increasing, and the outcomes of these procedures are superior to those of the United Network for Organ Sharing in both the short and long term, by reducing immunologic risk. Positive results from KEPs have been reported not only in Western countries but also in India [45,46] and Turkey [47,48]. Thus, KEPs are expected not only to expand transplant opportunities but also to improve transplant outcomes by avoiding immunological risks [40,41,49,50]. While the global introduction of KEPs has been advocated, ethical issues, such as systemic differences between countries and income disparities, have also been raised as challenges [51] of the disease.

Although this study conducted virtual cross-matching by collecting information only on cases in which transplantation was avoided due to immunological high-risk, we believe that if it were possible to avoid DSA and achieve successful transplantation without the need for desensitization therapy, the risk of infection and other complications associated with transplantation could be further reduced. Scurt et al. [52] conducted a meta-analysis of blood type-incompatible transplants and reported worse outcomes and safety compared with compatible transplants without desensitization therapy. Aida et al. [53] also reported a sustained decrease in the B-cell fraction with the use of rituximab at the time of transplantation as a risk factor for severe COVID-19 infection. Thus, avoiding desensitization therapy and using a KEP to enable transplantation will help reduce the risk of infection and other complications after transplantation.

Building on this rationale, expanding the benefits of KEP to a broader population of immunologically high-risk kidney transplant candidates ultimately depends on the establishment of an effective operational framework capable of enlarging the donor pool. The scope and success of such a program are heavily influenced by specific policy decisions—such as whether ABO-incompatible transplantation is permitted or systematically avoided, the selection of assays used to detect DSAs, and the thresholds set for excluding candidates based on immunological risk. Additionally, the geographic scale of a KEP—whether regional, national, or international—directly impacts the size and diversity of the donor pool.

Experiences from the United States demonstrate that, beyond conventional one-to-one paired exchanges, innovative strategies such as chain exchanges and voucher systems can significantly increase transplantation opportunities [32,35,36]. These models highlight how thoughtful program design can not only expand the donor pool but also better serve candidates likely to require future transplants. In light of the current study’s findings, which emphasize the challenges faced by immunologically high-risk recipients in identifying compatible donors, integrating such strategies could meaningfully enhance equity and access in the Japanese context. Future research should focus on assessing the feasibility, cost-effectiveness, and ethical considerations of implementing these models within Japan’s healthcare system, as well as the logistical and policy adaptations required for their successful deployment.

However, as the primary research suggests significant ethical concerns, we believe that when introducing a KEP in Japan, it may be beneficial to begin with one-on-one paired-exchange kidney transplants and initially conduct an ethical review of each patient pair in the early stages of the disease. There are various system-building issues to be considered, such as which hospital should perform the kidney harvesting procedure and transport the kidney graft? In addition, will the pairings occur on the same day? If the donation is not on the same day, what will happen if a donor who is scheduled to donate at a later date withdraws his/her intention to donate? There are various system-building challenges to consider.

Twenty years ago, the Japan Society for Transplantation stated that although exchange kidney transplantation does not violate its ethical guidelines, many transplant specialists believe that there is no overwhelming medical necessity for performing transplants through donor exchange. They also stated that social systems, such as donor exchange networks, should not be used to encourage donor exchange kidney transplantation. However, the present study demonstrated that there is a certain need for KEPs because approximately 25 candidate pairs per year are excluded from kidney transplantation due to immunological high-risk, and roughly half of these candidates could benefit from transplantation via a KEP. In addition, more than 60% of kidney transplant facilities believed that a KEP was necessary. Kidney exchange transplantation through personal communication within or between individual facilities tends to be rather unfair, and the creation of a nationwide KEP would be a more ethically fair system. It is also clear from reports from various researchers that the larger the donor pool, the higher the likelihood of transplantation opportunities for highly sensitized recipients. Based on the results of the present study, we hope to deepen the discussion on the pros and cons of establishing a KEP in Japan and the nature of such a KEP system.

## 5. Limitations

This study has many limitations, particularly in the simulation using virtual cross-matching (secondary research). First, the patients were enrolled based on a retrospective chart review of cases in which transplantation was abandoned owing to high immunological risks. The enrolled patients are currently being followed-up at dialysis facilities, rather than transplantation facilities, and the information available up to the time of the transplantation abandonment was limited. In Japan, the cost of various donor tests, including cross-matching, is covered by the national health insurance program only when the transplant has been successfully established. If the transplant has not been successfully established, the cost of all tests, including the anti-HLA antibody single antigen identification test, is entirely borne by the patient. For this reason, further immunological examinations and other donor tests are not often performed after a positive cross-match result and transplantation is abandoned. Therefore, sufficient information for virtual cross-matching simulation was available only for a small number of patients, and the cohort used for simulation represented approximately one-third of the patients for whom transplantation was abandoned.

Additionally, the data on potential donors and recipients were collected in a metachronous manner. Since the past decade, desensitization therapy options, such as rituximab and IVIG, are being covered by the national health insurance program; however, the exclusion criteria differ from center to center with changes being made over time. Thus, it was difficult to standardize the exclusion criteria. Therefore, many facilities cited a positive direct T-warm cross-match as a criterion for transplantation abandonment. However, various cross-match results actually led to a transplantation being avoided.

Moreover, the manufacturers of the anti-HLA antibody single antigen identification tests have not been standardized. Thus, a similar virtual cross-matching simulation study with a prospective design should be considered in the future. Furthermore, we used a cumulative sum of negative cases in virtual cross-matching of KEP donor candidates, without considering the overlap of cases. Ideally, the feasibility of KEP should be further examined in detail by considering how often KEP is possible on a one-on-one basis without the overlapping of donor candidates as well as the fairness-related factors such as donor age and renal function.

## 6. Conclusions

In Japan, 251 pairs of living-donor kidney transplants were canceled because of immunological high-risk over the 10-year period from 2012 to 2021. The results of HLA typing and anti-HLA antibody single antigen identification tests of 80 pairs showed that a KEP could be established in approximately half of the pairs without DSA, indicating the need for and potential benefits of a KEP in Japan. Going forward, further in-depth discussions on the potential issues in actual KEP implementation and the associated ethical issues are needed for the establishment of KEPs in Japan.

## Figures and Tables

**Table 1:** Comparison of donor candidate backgrounds.

Group		Overall Secondary Research Enrollment	Virtual Cross-Match Group	Excluded Group	*p*-Value *	Living Kidney Transplantation (2012–2021)	*p*-Value **
n		117	80	37		14,354	
Age (years)		56.5 ± 12.1	56.3 ± 11.0	56.9 ± 14.5	0.815	58.0	N/A
Age distribution	20–29	1	0	1	**0.026**	128	0.138
	30–39	6	5	1		672	
	40–49	20	16	4		2314	
	50–59	38	31	7		4003	
	60–69	39	20	19		4862	
	70–79	9	7	2		1919	
	80-	2	1	1		75	
Sex (male/female)		81:35	58:22	23:13	0.386	5213:9141	**<0.001**
Relationship (spouse(husband/wife)/parent/sibling/other)		81(70:11):17:13:6	59(51:8):11:6:4	22(19:3):6:7:2	0.131	5589:4885:1433:2447	**<0.001**
Hypertension (yes/no)		20:89	17:61	3:28	**0.018**	2481:9202	0.890
Diabetes mellitus (yes/no)		10:101	9:69	1:32	0.058	568:11,109	**0.014**
s-Cre (mg/dL)		0.85 ± 0.62	0.82 ± 0.14	0.93 ± 1.15	0.430	0.70	N/A
eGFR (mL/min/1.73 m^2^)		74.4 ± 14.5	72.6 ± 12.3	78.3 ± 18.4	0.074	N/A	N/A

* Comparison between the virtual cross-match group and the excluded group. ** Comparison between the virtual cross-match group and living kidney transplantation (2012–2021). N/A, not available. Bolded numbers indicate significant differences.

**Table 2:** Comparison of recipient candidate background.

		Overall Secondary Research Enrollment	Virtual Cross-Match Group	Excluded Group	*p* Value *	Living Kidney Transplantation (2012–2021)	*p* Value **
		117	80	37		14,354	
Age (years)		52.1 ± 10.6	51.2 ± 10.7	53.9 ± 10.3	0.201	47.2	N/A
Age distribution	0–9	1	1	0	**0.166**	252	1.000
	10–19	0	0	0		430	
	20–29	1	1	0		1156	
	30–39	18	10	8		2388	
	40–49	27	23	4		3415	
	50–59	46	31	15		3199	
	60–69	24	14	10		2922	
	70-	0	0	0		592	
Sex (male/female)		22:95	15:65	7:30	1.000	9192:5162	**<0.001**
Blood type (compatible/incompatible)		65:49	45:35	20:14	0.839	8671:4033	**0.029**
HLA mismatch	0	2	0	2	**0.024**	535	**0.024**
	1	0	0	0		682	
	2	6	2	4		2099	
	3	30	17	13		3458	
	4	20	16	4		1607	
	5	41	30	11		1743	
	6	18	15	3		953	
Direct XM T warm (positive/negative)		50:54	32:39	18:15	0.669	81:11,248	**<0.001**
Direct XM B warm (positive/negative)		58:39	37:30	21:9	**0.044**	265:11,007	**<0.001**
Flow XM T (positive/negative)		86:21	61:14	25:7	0.717	501:11,026	**<0.001**
Flow XM B (positive/negative)		102:2	73:0	29:2	0.083	990:10,147	**<0.001**
Flow PRA class1	0–less than 20%	9	6	3	0.847	5276	**<0.001**
	20–less than 40%	6	3	3		180	
	40–less than 60%	8	6	2		113	
	60–less than 80%	9	7	2		70	
	80–100%	17	12	5		66	
Flow PRA class2	0–less than 20%	13	12	1	0.101	5334	**<0.001**
	20–less than 40%	2	2	0		134	
	40–less than 60%	9	6	3		72	
	60–less than 80%	6	6	3		60	
	80–100%	15	7	8		66	
DSA (positive/negative)		117:0	80:0	37:0	1.000	777:5837	**<0.001**

* Comparison between the virtual cross-match group and the excluded group. ** Comparison between the virtual cross-match group and living kidney transplantation (2012–2021). N/A, not available. Bolded numbers indicate significant differences.

## Data Availability

The data that support the findings of this study are available from the corresponding author upon reasonable request.

## Abbreviations

The following abbreviations are used in this manuscript:

DSA	donor-specific antibody
eGFR	estimated glomerular filtration rate
HLA	human leukocyte antigen
KEP	kidney exchange program
N/A	not available
s-Cre	serum creatinine level
XM	cross-match
